# Supplementary figures and images for: Dairy Product Intake and Cardiometabolic Diseases in Northern Sweden: A 33-Year Prospective Cohort Study
Source: Nutrients. 2019 Jan 28;11(2):284. doi: 10.3390/nu11020284 (PMC6412239; doi:10.3390/nu11020284)

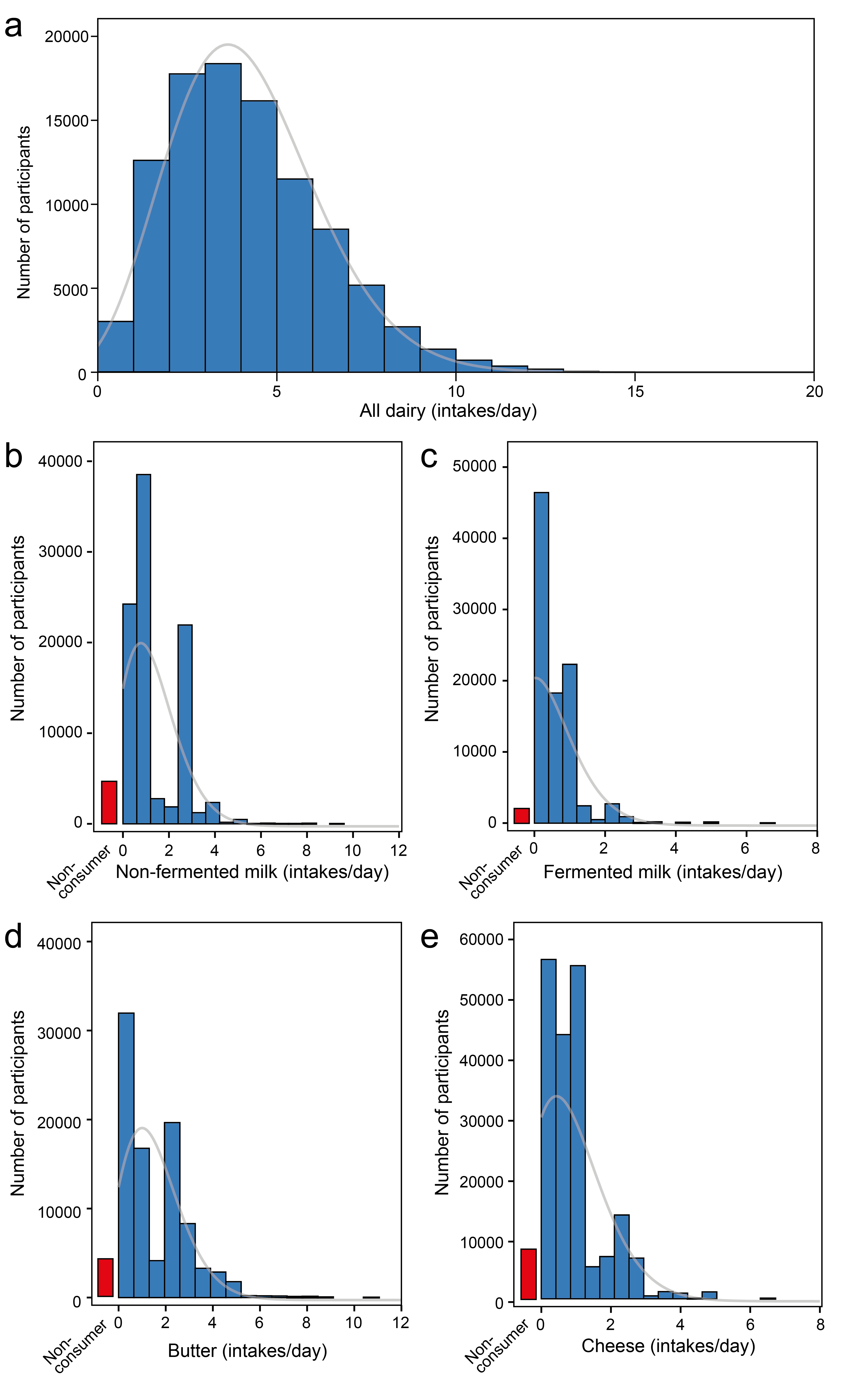

Supplement: Supplementary file 1 [file nutrients-11-00284-s001.zip › Supplementary files/Supplementary Figure S1.tif]
