# Supplementary material for: Dairy Product Intake and Cardiometabolic Diseases in Northern Sweden: A 33-Year Prospective Cohort Study
Source: Nutrients. 2019 Jan 28;11(2):284. doi: 10.3390/nu11020284 (PMC6412239; doi:10.3390/nu11020284)
Supplement: Supplementary file 1 [file nutrients-11-00284-s001.zip › Supplementary files/Supplementary Table S1.doc]

**Table S1. Proportions of non-consumers of targeted dairy products and intakes among consumers in incident type 2 diabetes (TD2), myocardial infarction (MI) and stroke groups and non-cases. Data are presented as sex, age and screening year adjusted means of intakes per day with (95% CI). Differences between the groups are all highly significant (p<0.001) for all variables.**

|  | Non- | Intake in consumers mean (95% CI | | | |
| --- | --- | --- | --- | --- | --- |
|  | consumers % | Non-cases (n=86,931) | TD2 (n=4,295) | Stroke  (n=3,692) | MI  (n=3,654) |
| **Dairy all types** | **0.2** | **4.15 (4.13, 4.16)** | **4.04 (3.97, 4.11)** | **4.15 (4.08, 4.22)** | **4.19 (4.12, 4.26)** |
| Q1 (range 2.80 intakes/day) |  | 1.69 (1.68, 1.70) | 1.65 (1.60, 1.71) | 1.72 (1.67, 1.78) | 1.74 (1.68, 1.79) |
| Q2 (range 1.94 intakes/day) |  | 3.18 (3.16, 3.19) | 3.22 (3.16, 3.28) | 3.22 (3.16, 3.28) | 3.26 (3.21, 3.32) |
| Q3 (range 2.45 intakes/day) |  | 4.60 (4.59, 4.61) | 4.62 (4.56, 4.68) | 4.62 (4.56, 4.68) | 4.67 (4.61, 4.72) |
| Q4 (range 14.2 intakes/day) |  | 7.12 (7.11, 7.13) | 7.16 (7.10, 7.22) | 7.10 (7.03, 7.15) | 7.14 (7.09, 7.20) |
| **Non-fermented milk** | **5.0** | **1.30 (1.29, 1.31)** | **1.40 (1.37, 1.44)** | **1.37 (1.34, 1.41)** | **1.40 (1.37, 1.43)** |
| Q1 (range 0.70 intakes/day) |  | 0.23 (0.22, 0.23) | 0.22 (0.18, 0.25) | 0.22 (0.19, 0.25) | 0.23 (0.20, 0.25) |
| Q2 (range 0.70 intakes/day) |  | 0.87 (0.86, 0.87) | 0.88 (0.85, 0.91) | 0.88 (0.86, 0.91) | 0.88 (0.86, 0.91) |
| Q3 (range 1.50 intakes/day) |  | 1.38 (1.37, 1.39) | 1.27 (1.24, 1.31) | 1.25 (1.22, 1.28) | 1.32 (1.29, 1.35) |
| Q4 (range 10.0 intakes/day) |  | 2.81 (2.81, 2.82) | 2.82 (2.79, 2.85) | 2.77 (2.75, 2.79) | 2.78 (2.76, 2.81) |
| **Fermented milk** | **4.8** | **0.60 (0.60, 0.61)** | **0.57 (0.55, 0.58)** | **0.54 (0.53, 0.56)** | **0.55 (0.53, 0.56)** |
| Q1 (range 0.22 intakes/day) |  | 0.08 (0.07, 0.08) | 0.08 (0.07, 0.10) | 0.08 (0.07, 0.10) | 0.08 (0.07, 0.09) |
| Q2 (range 0.56 intakes/day) |  | 0.28 (0.28, 0.28) | 0.30 (0.28, 0.32) | 0.31 (0.30, 0.33) | 0.29 (0.28, 0.31) |
| Q3 (range 0.64 intakes/day) |  | 0.70 (0.69, 0.7) | 0.74 (0.72, 0.76) | 0.75 (0.74, 0.77) | 0.71 (0.69, 0.72) |
| Q4 (range 7.28 intakes/day) |  | 1.31 (1.30, 1.31) | 1.371 (1.35, 1.39) | 1.30 (1.28, 1.31) | 1.30 (1.29, 1.32) |
| **Butter** | **9.1** | **1.52 (1.51, 1.53)** | **1.50 (1.45, 1.55)** | **1.58 (1.53, 1.63)** | **1.61 (1.56, 1.65)** |
| Q1 (range 0.36 intakes/day) |  | 0.05 (0.05, 0.06) | 0.05 (0.01, 0.09) | 0.06 (0.02, 0.09) | 0.06 (0.02, 0.09) |
| Q2 (range 2.28 intakes/day) |  | 0.66 (0.66, 0.67) | 0.67 (0.63, 0.72) | 0.68 (0.64, 0.71) | 0.73 (0.69, 0.77) |
| Q3 (range 1.58 intakes/day) |  | 1.98 (1.98, 1.99) | 2.15 (2.11, 2.20) | 2.18 (2.14, 2.22) | 2.26 (2.23, 2.29) |
| Q4 (range 9.50 intakes/day) |  | 3.33 (3.32, 3.34) | 3.45 (3.41, 3.49) | 3.35 (3.31, 3.39) | 3.47 (3.44, 3.51) |
| **Cheese** | **2.7** | **0.98 (0.97, 0.99)** | **0.91 (0.88, 0.94)** | **0.95 (0.92, 0.97)** | **0.92 (0.89, 0.95)** |
| Q1 (range 0.44 intakes/day) |  | 0.22 (0.21, 0.23) | 0.21 (0.18, 0.24) | 0.21 (0.18, 0.25) | 0.22 (0.19, 0.25) |
| Q2 (range 0.48 intakes/day) |  | 0.59 (0.59, 0.60) | 0.58 (0.55, 0.62) | 0.60 (0.56, 0.63) | 0.58 (0.55, 0.61) |
| Q3 (range 0.42 intakes/day) |  | 0.98 (0.97, 0.98) | 0.98 (0.95, 1.01) | 0.98 (0.94, 1.01) | 0.97 (0.94, 1.00) |
| Q4 (range 6.94 intakes/day) |  | 2.10 (2.09, 2.10) | 2.02 (1.99, 2.05) | 2.09 (2.06, 2.13) | 2.03 (2.00, 2.05) |
| **Non-fermented milk, 0.5% fat** | **55.5** | **0.67 (0.66, 0.68)** | **0.81 (0.76, 0.85)** | **0.74 (0.70, 0.79)** | **0.74 (0.70, 0.78)** |
| **Non-fermented milk, 3% fat** | **55.1** | **0.36 (0.36, 0.37)** | **0.40 (0.36, 0.44)** | **0.48 (0.44, 0.52)** | **0.44 (0.40, 0.47)** |
| **Fermented milk, 0.5% fat** | **24.9** | **0.27 (0.27, 0.27)** | **0.29 (0.27, 0.30)** | **0.25 (0.23, 0.26)** | **0.25 (0.24, 0.27)** |
| **Fermented milk, 3 % fat** | **9.4** | **0.41 (0.40, 0.41)** | **0.36 (0.35, 0.38)** | **0.38 (0.36, 0.40)** | **0.37 (0.36, 0.39)** |
| **Cheese 10-17% fat** | **27.3** | **0.39 (0.38, 0.39)** | **0.39 (0.37, 0.42)** | **0.38 (0.36, 0.41)** | **0.38 (0.36, 0.40)** |
| **Cheese 28 % fat** | **9.7** | **0.74 (0.73, 0.74)** | **0.66 (0.64, 0.69)** | **0.72 (0.69, 0.75)** | **0.70 (0.67, 0.72)** |
